# Molasses-amended siderophore biofertilizer reshapes the rhizosphere microbiome, promotes growth and reduces lipid peroxidation in *Ocimum basilicum* L

**DOI:** 10.3389/fpls.2026.1879696

**Published:** 2026-08-13

**Authors:** Marcin Musiałowski, Anna Bernatowicz, Łucja Kowalewska, Aleksandra Goszcz, Xiaomeng Chen, Silvia Celletti, Michela Schiavon, Klaudia Dębiec-Andrzejewska

**Affiliations:** 1 Laboratory of Agricultural and Industrial Microbiology, Institute of Bioengineering, Faculty of Biology, University of Warsaw, Warsaw, Poland; 2 Department of Plant Anatomy and Cytology, Faculty of Biology, Institute of Experimental Plant Biology and Biotechnology, University of Warsaw, Warsaw, Poland; 3 Department of Agricultural, Forest and Food Sciences (DISAFA), University of Turin, Turin, Italy

**Keywords:** basil, oxidative stress alleviation, photosynthetic efficiency, plant biostimulants, plant-microbe interaction, psychrotolerant bacteria, soil biostimulation

## Abstract

**Introduction:**

Siderophore-producing bacteria and their metabolites represent promising components of next-generation biofertilizers, yet their effects on plant physiology and soil microbiome structure remain insufficiently understood.

**Methods:**

We developed a liquid biofertilizer based on siderophores and siderophore-accompanying metabolites (SSAM) produced by *Pseudomonas* sp. ANT_H12B and formulated with molasses as an organic carrier. Its effects on sweet basil (*Ocimum basilicum* L.) were evaluated by assessing plant growth, photosynthetic performance, lipid peroxidation, elemental composition, soil enzyme activities, and bacterial community structure using full-length 16S rRNA nanopore sequencing.

**Results:**

The combined SSAM+molasses formulation significantly enhanced plant growth, increasing leaf dry biomass by nearly 180%, leaf number by more than 300%, and stem length by approximately 40–50% compared with untreated plants. Improved plant performance was accompanied by enhanced photosynthetic efficiency (F_v_/F_M_) and a marked reduction in oxidative stress, as reflected by nearly 50% lower malondialdehyde (MDA) content compared with the molasses-only treatment. Although elemental analysis revealed no major disturbances in plant nutrient balance among treatments, soil supplementation with the combined formulation strongly affected rhizosphere functioning and microbiome composition. In particular, the SSAM+molasses treatment coincided with approximately 35–50% higher β-glucosidase and dehydrogenase activities and clear shifts in microbial community structure.

**Discussion:**

These findings suggest that molasses-enriched siderophore metabolites may act as effective biostimulants by promoting plant growth and mitigating oxidative stress, partly through modulation of rhizosphere microbiome structure and function.

## 1 Introduction

The growing demand for sustainable agricultural practices and the increasing awareness of the environmental consequences of intensive fertilization have driven the search for alternative strategies to improve crop productivity while preserving soil health and ecosystem stability. Conventional synthetic fertilizers, although effective in increasing yields, are often characterized by low nutrient-use efficiency and can negatively affect soil physicochemical properties and microbial diversity (Timilsena et al., 2015; Cai et al., 2019). Their excessive application may lead to nutrient imbalances, soil degradation, and disruption of beneficial plant-microbe interactions (Savci, 2012; Chen et al., 2008; Geisseler and Scow, 2014; Sabry, 2015). Consequently, biofertilizers and biostimulants based on plant growth-promoting bacteria (PGPB) and their biologically active metabolites have emerged as promising tools for improving plant productivity and stress resilience in a more sustainable manner (Malusá and Vassilev, 2014; Mahanty et al., 2016; Chaudhary et al., 2022; Shah et al., 2024).

Many biofertilizers rely on the application of live microorganisms, but increasing attention has been recently directed toward the use of bacterial metabolites as active biostimulatory agents (Chaudhary et al., 2022). These metabolites include phytohormones, organic acids, exopolysaccharides, volatile compounds, and siderophores, which can modulate nutrient availability and plant metabolism, while also shaping plant-microbial interactions in the rhizosphere (Glick, 2012; Mahanty et al., 2016; Allouzi et al., 2022). Through these functions, bacterial metabolites can regulate root development, enhance nutrient acquisition, and modulate stress tolerance (Bacilio et al., 2004; Fuentes-Ramirez and Caballero-Mellado, 2005; Chaudhary et al., 2022; Gahlot, 2026). Beyond direct effects on plants, they also influence soil biochemical processes and microbial community dynamics, contributing to improved soil fertility and functional plant-microbiome associations (Gupta et al., 2022). In this context, microbial metabolites may act as ecological drivers that selectively favor beneficial microbial taxa, thereby enforcing plant-microbiome cooperation and ultimately supporting improved plant performance (Dong et al., 2025; Nie et al., 2025).

Among bacterial metabolites, siderophores play a particularly important role in plant-soil systems. Siderophores are low-molecular-weight iron (Fe)-chelating compounds produced by many soil bacteria, especially species belonging to the genus *Pseudomonas* (Hider and Kong, 2010; Goswami et al., 2016; Musialowski et al., 2023). Fe is an essential micronutrient required for photosynthesis, respiration, and numerous enzymatic processes, but its availability in soil is often limited due to its presence in insoluble ferric forms (Hider and Kong, 2010; Saha et al., 2013). Siderophores chelate Fe³^+^ and facilitate its solubilization and uptake by plants, thereby improving plant Fe nutrition and physiological performance (Robin et al., 2008; Radzki et al., 2013). In addition, they facilitate the uptake and mobility of other essential nutrients (i.e., copper (Cu), zinc (Zn), manganese (Mn)), thereby contributing to plant biofortification. At the same time, siderophores can mitigate metal-induced stress by immobilizing potentially phytotoxic elements (Gamalero and Glick, 2011; Olanrewaju et al., 2017).

Beyond their direct effects on plant nutrition and physiology, siderophores and other bacterial metabolites also play a relevant ecological role in structuring soil microbial communities (Nie et al., 2025). The rhizosphere represents a highly dynamic environment where microbial metabolites, root exudates, and microbial interactions jointly shape microbial community composition and activity (Compant et al., 2010; Gupta et al., 2022). These interconnected processes regulate nutrient cycling, enzyme activity, and overall soil fertility. Accordingly, microbial metabolites can function not only as plant biostimulants but also as key regulators of rhizosphere microbiome assembly and function, with broader implications for plant-microbe interactions and ecosystem processes (Trivedi et al., 2020).

The effectiveness of metabolite-based biofertilizers depends not only on the metabolites themselves, but also on their formulation and delivery (Malusá et al., 2012). Organic carriers play a critical role in improving the application efficiency of microbial metabolites by facilitating their distribution in soil and providing additional carbon (C) sources for soil microorganisms (Malusá et al., 2012; Dzionek et al., 2016). Among these carriers, molasses (a by-product of sugar production rich in simple sugars, organic compounds, and mineral nutrients) has emerged as a promising option owing to its biodegradability, low production cost, and high nutritional value (Olbrich, 1963; Garcha et al., 2019; Lal, 2020; Li et al., 2020). Molasses can stimulate microbial activity, improve soil physicochemical properties, and enhance plant growth and nutrient availability (Jiang et al., 2012; Li et al., 2020). Because molasses provides readily degradable carbon and mineral nutrients, it may actively influence rhizosphere microbial activity and plant physiological responses rather than function solely as an inert delivery matrix. Nutrient-rich by-products of the sugar and distillery industries may also act as biologically active soil amendments; for example, digested distillery spent wash improved nodulation, nutrient uptake, and photosynthetic activity in chickpea (Gahlot et al., 2011).

Although siderophores and molasses have individually been investigated as components of plant biostimulants and biofertilizers, their combined effects on plant physiology and soil microbial communities remain insufficiently understood, particularly whether molasses independently contributes to these responses or modifies the activity of the metabolite fraction. To address this gap, we evaluated a cell-free fraction containing siderophores and siderophore-associated metabolites (SSAM) produced by *Pseudomonas* sp. ANT_H12B, applied with or without molasses, using a two-factor design that separated the individual and interactive effects of both components.

Basil (*Ocimum basilicum* L. var. genovese) was used as a model plant species because of its relatively high micronutrient demand and clear physiological responses, including chlorosis and growth changes, under Fe limitation over short experimental periods. We evaluated plant growth, photosynthetic performance, oxidative stress status, and elemental composition. In parallel, we investigated soil enzymatic activity and bacterial community structure in the rhizosphere and bulk soil using full-length 16S rRNA gene sequencing. We hypothesized that combining SSAM with molasses would enhance basil growth and photosynthetic performance, reduce lipid peroxidation, and modify rhizosphere enzymatic activity and bacterial community structure relative to the untreated control and the individual components.

## 2 Materials and methods

### 2.1 Bacterial strain, plant seeds and soil

The bacterial strain used in this study was *Pseudomonas* sp. ANT_H12B (GenBank assembly accession number: GCA_008369325.1), isolated from Antarctic soil samples collected at King George Island (Antarctica; GPS coordinates: 62°09.6010′ S, 58°28.4640′ W). This psychrotolerant strain has been previously genetically and metabolically characterized, including optimization of siderophore production (Musialowski et al., 2023; Styczynski et al., 2022). It has been reported to produce more than 500 μM of pyoverdine, confirming its high siderophore production capacity, as well as other plant growth-promoting metabolites, including indole-3-acetic acid (IAA) (Musialowski et al., 2023).

Seeds of sweet basil were used in the plant experiment. The seeds were obtained from the Breeding and Seed Center POLAN (Poland). Plants were cultivated in 800-mL pots. Prior to use, the soil was homogenized and sieved through a 2-mm mesh without sterilization. The soil exhibited moderately acidic conditions (pH 6.4) and low salinity (0.38 mS cm^−1^). It was characterized by a relatively high organic matter content, with organic C (C_org) of 6.25% and total carbon (TC) concentration of 69,872 mg kg^−1^ dry matter. Total nitrogen (TN) content was 7,631 mg kg^−1^ dry matter and soil moisture content was 18.65%. In terms of nutrient composition, potassium and magnesium occurred at similarly high concentrations (4,379 and 4,044 mg kg^−1^, respectively), whereas calcium was detected at a markedly lower level (264 mg kg^−1^), with sodium remaining comparatively low (293 mg kg^−1^).

### 2.2 Bacteria cultivation and siderophore production

To exploit the full metabolic potential of bacterial metabolites, siderophores and SSAM produced by *Pseudomonas* sp. ANT_H12B were used without purification, as previously demonstrated to enhance plant growth (Musialowski et al., 2023, 2026). The strain was cultivated overnight in lysogeny broth (LB) medium at 20 °C with rotary shaking at 150 rpm. Subsequently, 50 mL of the culture was centrifuged (8,000 rpm, 5 min), washed twice with 0.85% NaCl solution to remove residual medium components, and resuspended in 50 mL of the same solution to obtain the inoculum. The biomass suspension was used to inoculate GCl medium (5.25 g L^−1^ glucose, 1.35 g L^−1^ NH_4_Cl, 0.96 g L^−1^ Na_2_HPO_4_, 0.44 g L^−1^ KH_2_PO_4_, and 0.2 g L^−1^ MgSO_4_×7H_2_O). Cultures were incubated for 48 h at 10 °C with rotary shaking at 150 rpm, according to Musialowski et al. (2023). Following incubation, cultures were centrifuged (8,000 rpm, 8 min), and the supernatants were filtered through 0.22 µm syringe filters to remove residual cells. The resulting sterile supernatants contained siderophores (primarily pyoverdine) along with co-produced metabolites (SSAM), including IAA, and were used for further experiments. The detailed composition of SSAM according to Musialowski et al., 2023 was presented in **Table 1**.

**Table 1:** The chemical properties of SSAM produced by *Pseudomonas* sp. ANT_H12B and potential biological relevance of parameters.

Parameter	Value	Potential biological relevance
pH	5.5	Acidity of the metabolite preparation
EC	101.6 µS/cm	Ionic strength of the preparation
C_total_	1210 mg/L	Overall carbon content
C_org_	1190 mg/L	Potential carbon input for rhizosphere microorganisms
N	296 mg/L	Nutrient supply and microbial metabolism
P	260 mg/L	Plant nutrition and microbial metabolism
K	141 mg/L	Osmotic regulation and plant nutrition
Mg	18.1 mg/L	Enzyme function and photosynthetic metabolism
S	23.0 mg/L	Sulfur metabolism and redox-related processes
Organic acids	80.1 mg/L	Potential contribution to nutrient solubilization, chelation, and rhizosphere pH modification
Sugars	0.17 mg/L	Readily available carbon compounds
Indolic acid (IAA)	2.5 μm/L	Regulation of root development and plant growth
Alcohols	7.7 mg/L	Microbial metabolic constituents
Esters	7.6 mg/L	Microbial metabolic constituents
Siderophores concentration (pyoverdine)	500 μM	Fe chelation and potential modification of Fe availability at the root–soil interface

### 2.3 Production of biofertilizer formulations

The obtained siderophore-containing supernatant was combined with beet molasses used as the organic carrier (Pfeifer & Langen, Poland). Molasses was selected to biochemically stabilize bacterial metabolites by increasing osmotic pressure and to provide an additional source of organic C for plants and soil microorganisms. Prior to formulation, the individual components were characterized physicochemically. The molasses had a pH of 6.31, an electrical conductivity (EC) of 21.12 mS cm^−1^, and a total organic carbon (TOC) content of 76.07% (w/v), whereas the SSAM had a pH of 5.50, an EC of 0.102 mS cm^−1^, and a TOC content of 1.19% (w/v). Based on preliminary studies, a biofertilizer formulation consisting of molasses:SSAM at a ratio of 2:8 (v/v) was prepared for further experimental stages. The formulation was freshly prepared prior to each application. Its physicochemical properties, including pH, EC, TOC, and siderophore concentration, were determined prior to use to ensure consistency and to relate formulation characteristics to plant responses. The final formulation was moderately acidic, with a pH of 5.96, an EC of 4.11 mS cm^−1^, and a TOC content of 19.83% (w/v).

### 2.4 Plant experimental setup

Basil plants were cultivated in 800-mL pots, containing a mixture of sandy soil and peat (1:1, v/v), supplemented with 15% (w/w) perlite to improve aeration. A total of 16 pots (1 plant/pot) were used in the experiment, corresponding to four experimental treatments with four biological replicates each (n = 4). The treatments included: (i) distilled water (control), (ii) molasses, (iii) siderophore-containing supernatant (SSAM), and (iv) a combined formulation of SSAM and molasses (SSAM+molasses). The final treatments contained 2% (v/v) molasses, 8% (v/v) SSAM, or a combination of 2% (v/v) molasses and 8% (v/v) SSAM. Accordingly, each 50-mL treatment application supplied 1 mL of molasses and/or 4 mL of SSAM per plant. Plants were grown under controlled conditions (temperature: 25 °C, photoperiod 16/8 h, relative humidity: 63 ± 5%, and light intensity: 200 μM photons m^−2^ s^−1^). Pots were arranged in a completely randomized design and rotated after each watering event to minimize positional effects. Plants were watered twice weekly throughout the 7-week experiment, resulting in 14 watering 50 mL each. Treatment groups received seven alternating applications of the corresponding diluted treatment solution and deionized water, whereas control plants received deionized water at every watering event. Thus, all plants received a total of 700 mL of liquid, including 350 mL of diluted treatment solution in the treated groups, equivalent to a cumulative input of 7 mL of molasses and/or 28 mL of SSAM per plant. Based on the pyoverdine concentration of 500 μM in the original SSAM fraction, its final concentration in both SSAM-containing treatment solutions was 40 μM after formulation and dilution. Thus, each 50-mL treatment application delivered approximately 2 μmol of pyoverdine, corresponding to a cumulative input of 14 μmol per plant over seven applications.

### 2.5 Analysis of the morphometric traits, photosynthetic parameters of plants and lipid peroxidation

After 7 weeks of growth and treatment, basil plants were harvested and either processed immediately for selected analyses or stored at -80 °C for subsequent use. On the 49th day of growth, chlorophyll fluorescence was measured using the Imaging-PAM Maxi fluorometer (Heinz Walz, Germany). Leaf discs (1.5 cm diameter) were dark-adapted on moist blotting paper for 20 min before measurement. Minimal fluorescence (F_0_) was recorded under weak blue modulating light (0.5 μM photons m^−2^ s^−1^), and maximal fluorescence (F_M_) was induced with a saturating pulse (2,700 μM photons m^−2^ s^−1^, 0.84 s), as described in Mazur et al. (2021). Actinic light (450 μM photons m^−2^ s^−1^) was applied with periodic saturation pulses at 20 s intervals over 400 s. ImagingWinGigE software was used for data acquisition and analysis, following the protocol by Mazur et al. (2021). F_V_/F_M_ parameter was calculated according to the following formula: F_V_/F_M_ = (F_M_ – F_0_)/F_M_.

To accurately assess the impact of treatments on basil growth and phenotype, each plant was photographed before and after removal from the pot. After gentle washing and drying, shoot length, root length, and number of leaves were determined. Stem and root lengths were measured with a ruler to the nearest 1 mm. Stem length was measured from the root-shoot junction to the shoot apical meristem, while root length was measured from the base to the distal end of the longest lateral root. The leaf number on each plant was counted manually. Plants were then separated into roots, stems, and leaves. The fresh biomass (FW) of each plant part was weighed, after which samples were dried at 60 °C for 72 h and reweighed to register the dry weight (DW).

The malondialdehyde (MDA) assay was performed according to Dalla Vecchia et al. (2023). It is a product of lipid peroxidation determined *via* reaction with thiobarbituric acid. Its concentration was calculated from the absorbance at 532 nm (with correction for non-specific turbidity by subtracting absorbance at 600 nm) using an extinction coefficient of ϵ_532_ = 155 mM^−1^ cm^−1^. Malondialdehyde content was used as a quantitative indicator of lipid peroxidation intensity and, consequently, the extent of oxidative damage to cellular membranes in plant tissues.

### 2.6 Elemental analysis of soil and plant samples

At the end of the experiments, soil samples were collected from the bulk soil and the rhizosphere of each pot for further analysis. Soil samples were air-dried, homogenized, and sieved to ≤ 2 mm prior to analysis. Basic physicochemical properties, including pH and EC, were determined using standard procedures. Soil pH was measured in deionized water at a 1:2.5 (w/v) soil-to-solution ratio, while EC was assessed using a conductivity meter. Total carbon and TN were determined with an elemental analyzer (UNICUBE, Elementar Analysensysteme, GmbH, Langenselbold, Germany).

For elemental analysis, plant and soil samples were processed separately. Dried plant material was finely ground. Soil samples were similarly homogenized prior to digestion. All samples were subjected to microwave-assisted acid digestion using aqua regia (HCl/HNO_3_, 3:1 v/v; ETHOS D system, Milestone, Italy). The resulting digests were diluted with ultrapure water prior to analysis. Elemental concentrations, including potassium (K), phosphorus (P), sulfur (S) and Fe were quantified using Inductively Coupled Plasma-Mass Spectrometry (ICP-MS; NexION 350D, PerkinElmer, Shelton, USA). All measurements were expressed on a dry weight basis. Analytical quality was verified using certified reference materials and procedural blanks.

### 2.7 Analysis of microbial activity in soil

Soil microbial activity was assessed based on selected enzymatic indicators reflecting key biogeochemical processes. Enzyme activities were determined in both bulk soil and rhizosphere samples. Dehydrogenase activity (DHA), representing overall microbial oxidative activity, was determined using the iodonitrotetrazolium chloride (INT) reduction method according to Alef and Nannipieri (1995), with minor modifications. Briefly, 0.5 g of soil was mixed with 2 mL of 0.2% INT solution and incubated in the dark for 2 h under shaking conditions to allow reduction of INT to iodonitrotetrazolium formazan (INTF). Subsequently, 4 mL of an extractant mixture (dimethylformamide, 1:1 v/v) was added, and samples were incubated for an additional 1 h in the dark with intermittent mixing. After centrifugation (8,000 rpm, 2 min), 250 µL of the supernatant was transferred to a microplate, and absorbance was measured at 464 nm. β-glucosidase activity was determined using a colorimetric assay based on p-nitrophenol (pNP) release, following the standard protocol “SOP: Enzyme assays (pNP)” (Soils Lab, University of Illinois Urbana-Champaign, 2021a), Soils Lab, University of Illinois Urbana-Champaign (USA). Protease activity was assessed using a casein hydrolysis assay according to the protocol “SOP: Protease (casein) assay” (Soils Lab, University of Illinois Urbana-Champaign, 2021b), Soils Lab, University of Illinois Urbana-Champaign. All enzyme activities were expressed per gram of dry soil and measured in three independent replicates.

### 2.8 DNA extraction, amplification and sequencing from soil material

DNA was extracted from ~0.25 g bulk soil and rhizosphere samples using the DNeasy PowerSoil Pro Kit (QIAGEN, USA), with a DNA concentration measured by a Qubit4 Fluorometer with dsDNA HS Assay Kit. Bacterial 16S rRNA genes were PCR-amplified with primer pairs targeting 1492R-II (5’-CGG YTA CCT TGT TAC GAC TT-3’) and 27F-II (5’-AGRGTT YGATYM TGGCTC AG-3’) in a 25 μL reaction consisting of 0.5 μL KAPA HiFi HotStart, 5 μL KAPA HiFi buffer, 1 μL DNA template, 0.75 μL of dNTP mix, 0.75 μL of each primer, and 16.25 μL of nuclease-free water. Thermal cycling conditions for PCR amplification were as follows: initial denaturation at 95 °C for 3 min; 28 cycles of 95 °C for 30 s, 57 °C for 30 s, and 72 °C for 45 s, followed by a final elongation at 72 °C for 1 min. Obtained DNA material was purified using KAPA Pure Beads (Roche, Switzerland) and quantified with Qubit fluorometer. Sequencing libraries were prepared with the Native Barcoding Kit 96 v14 SQK-NBD114.96 (Oxford Nanopore Technologies, UK). Sequencing was carried out on a MinION Mk1C device using R10.4.1 flow cells.

### 2.9 Microbial community profiling

Basecalling and demultiplexing were performed using the Dorado basecaller. Reads were filtered by length (1200–1800 bp) using Chopper and quality-checked with NanoPlot (De Coster and Rademakers, 2023). Sequences were dereplicated and clustered using VSEARCH (Rognes et al., 2016), followed by taxonomic classification with Kraken2 against the SILVA SSU Ref NR 138.2 database (Quast et al., 2013; Wood et al., 2019). Alpha diversity analyses were conducted in R using the *vegan* package. Shannon, Simpson, and Pielou’s evenness indices were calculated using the diversity function. Prior to analysis, samples were rarefied to an equal sequencing depth using the *rrarefy* function, and rarefaction curves were generated with *rarecurve* to assess sampling completeness. For beta diversity analysis, relative abundance data were Hellinger-transformed using the *decostand* function. Community dissimilarities were calculated based on Bray-Curtis distances, and Non-metric Multidimensional Scaling (NMDS) ordination was performed using the *metaMDS* function. Environmental variables were fitted onto the NMDS ordination using the *envfit* function. Indicator species analysis was performed using the *multipatt* function from the *indicspecies* R package.

### 2.10 Statistical analysis

The experiment followed a complete 2 × 2 factorial design with molasses and SSAM as crossed factors, each at two levels (present/absent), and four biological replicates per treatment. Elemental composition, MDA, chlorophyll fluorescence, morphometric traits, and soil enzyme activities were analyzed using two-way ANOVA with Type III sums of squares and sum-to-zero contrasts. Main effects of molasses and SSAM and their interaction were tested, and effect sizes were expressed as partial eta-squared (η²p). Significant interactions were examined using simple main-effects tests based on the pooled error term of the full model. Full factorial output is given in **Supplementary Materials** as **Supplementary Table SM.1**, with simple effects data in Supplementary Materials as **Supplementary Table SM.2**. Differences among the four treatment groups were additionally evaluated by one-way ANOVA followed by Tukey’s HSD test at p ≤ 0.05. Multivariate differences in community structure were tested using PERMANOVA implemented in the adonis2 function in the vegan package. Statistical significance was determined at a p-value ≤ 0.05 (Oksanen et al., 2024). All analyses were conducted in RStudio 2025.09.2, with statistical significance set at p ≤ 0.05.

## 3 Results

### 3.1 Effects of siderophore-based biofertilizer on plants

**Figure 1:**
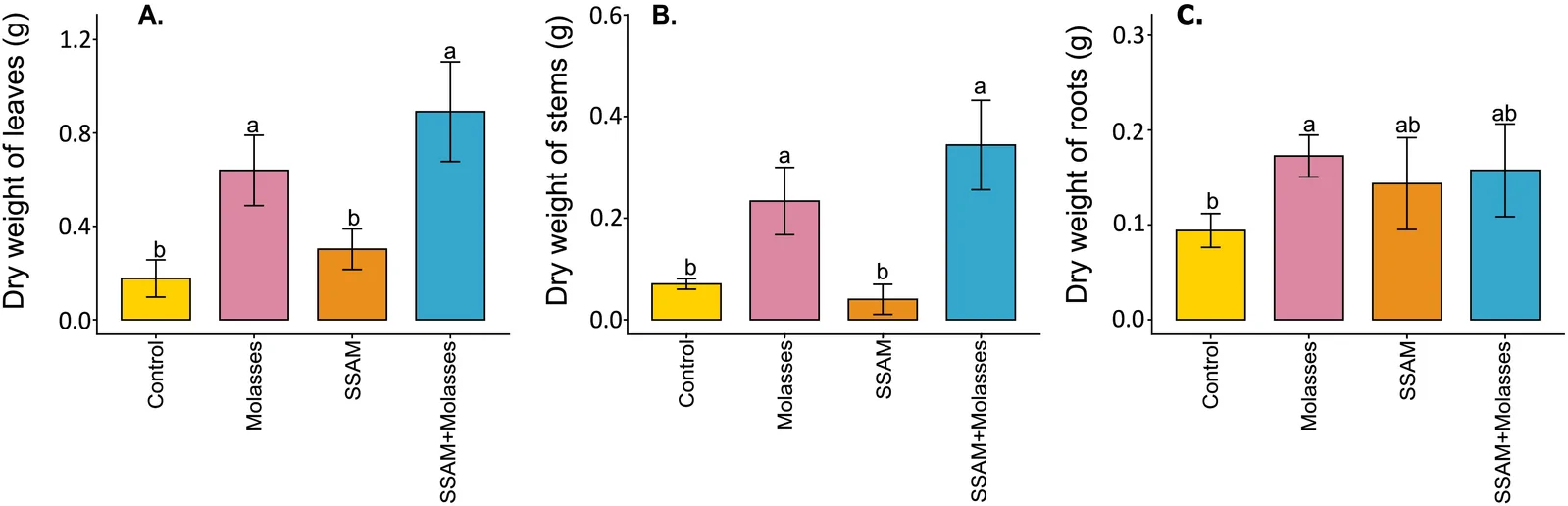
Dry biomass of basil leaves **(A)**, stems **(B)**, and roots **(C)** under control, molasses, SSAM, and SSAM + molasses treatments. Different letters indicate significant differences according to Tukey’s HSD test (p ≤ 0.05).

Plant growth in terms of DW biomass production reflected the observed traits (**Figure 1**). Leaf dry biomass was markedly enhanced by molasses-based treatments (ANOVA, p ≤ 0.001). In particular, plants supplied with SSAM+molasses accumulated nearly three times greater leaf biomass than control plants (0.89 ± 0.11 vs. 0.32 ± 0.05), while molasses alone resulted in an approximately twofold increase (0.64 ± 0.08). SSAM alone did not substantially alter leaf biomass compared to the control. In contrast, stem dry biomass remained relatively stable across treatments, with only a slight tendency toward higher values in the SSAM+molasses variant (0.34 ± 0.04). Root biomass showed only a limited response: the highest root dry mass was recorded in the molasses treatment (0.17 ± 0.01), corresponding to roughly a 40% increase relative to the control, while both SSAM-containing variants displayed intermediate values. Two-way factorial analysis revealed significant main effects of molasses (p < 0.001, η²p = 0.82) and SSAM (p = 0.022, η²p = 0.37) on leaf dry biomass, while the Molasses × SSAM interaction was not significant (p = 0.397). Stem dry biomass showed a significant Molasses × SSAM interaction (F = 6.03, p = 0.030, η²p = 0.34). Simple-effects analysis showed that molasses increased stem biomass by 0.304 g in the presence of SSAM (p < 0.001) and by 0.163 g in its absence (p = 0.002). Root dry biomass was significantly affected by molasses (p = 0.029, η²p = 0.34), whereas the Molasses × SSAM interaction was not significant (η²p = 0.20).

**Figure 2:**
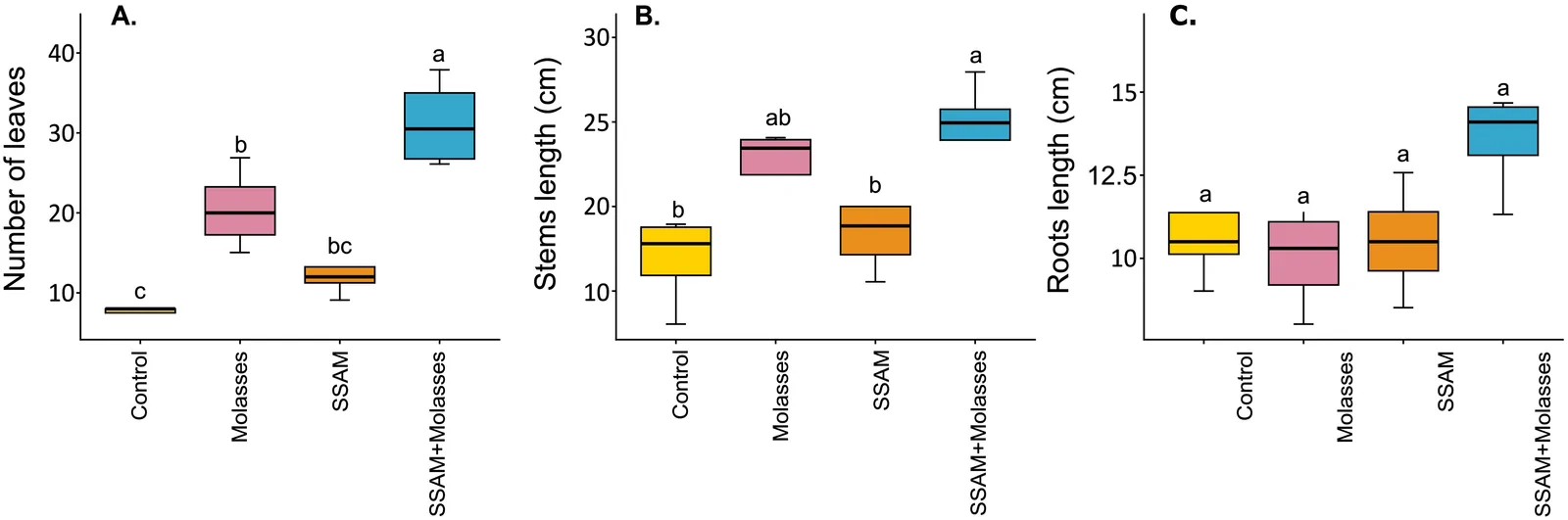
Number of leaves **(A)**, length of stems **(B)**, length of roots **(C)** of basil plants under control, molasses, SSAM, and SSAM + molasses treatments. Boxes show the interquartile range, horizontal lines indicate medians, and whiskers represent the data range. Different letters indicate significant differences according to Tukey’s HSD test (p ≤ 0.05).

The number of leaves was strongly affected by the treatment (p ≤ 0.001) (**Figure 2A**). The combined application of SSAM and molasses resulted in the most pronounced response, with plants developing on average more than four times as many leaves as the control (31.25 ± 2.87 vs. 7.50 ± 0.50, respectively). Molasses alone also substantially stimulated leaf formation, resulting in nearly a threefold increase compared to the control (20.50 ± 2.60). In plants treated with SSAM alone, the leaf number (12.50 ± 1.66) remained closer to the control group than to the molasses-based treatments. In the factorial model, leaf number responded to large main effects of both molasses (p < 0.001, η²p = 0.82) and SSAM (p = 0.003, η²p = 0.54), without a significant interaction (p = 0.200).

Stem length followed a similar pattern (**Figure 2B**). The longest stems were observed in the SSAM+molasses treatment (24.73 ± 1.43), exceeding both the control and the SSAM variant by approximately 40-50%. Molasses alone also promoted stem growth (22.38 ± 1.31) in comparison to the control, whereas SSAM treatment alone did not result in any clear improvement compared to untreated plants. Root length was not significantly affected by the treatment applied (**Figure 2C**). Although the highest mean root length was recorded in the SSAM+molasses variant (13.55 ± 0.78), variability among treatments was low overall, and no consistent trend emerged. Factorial analysis showed stem length to be governed exclusively by a main effect of molasses (p < 0.001, η²p = 0.63), with neither a significant SSAM effect (p = 0.178) nor an interaction (p = 0.723). Root length, in contrast, showed a significant Molasses × SSAM interaction (F = 5.28, p = 0.040, η²p = 0.31) in the absence of any significant main effect.

### 3.2 Elemental composition of plant tissues following biofertilizer application

**Figure 3:**
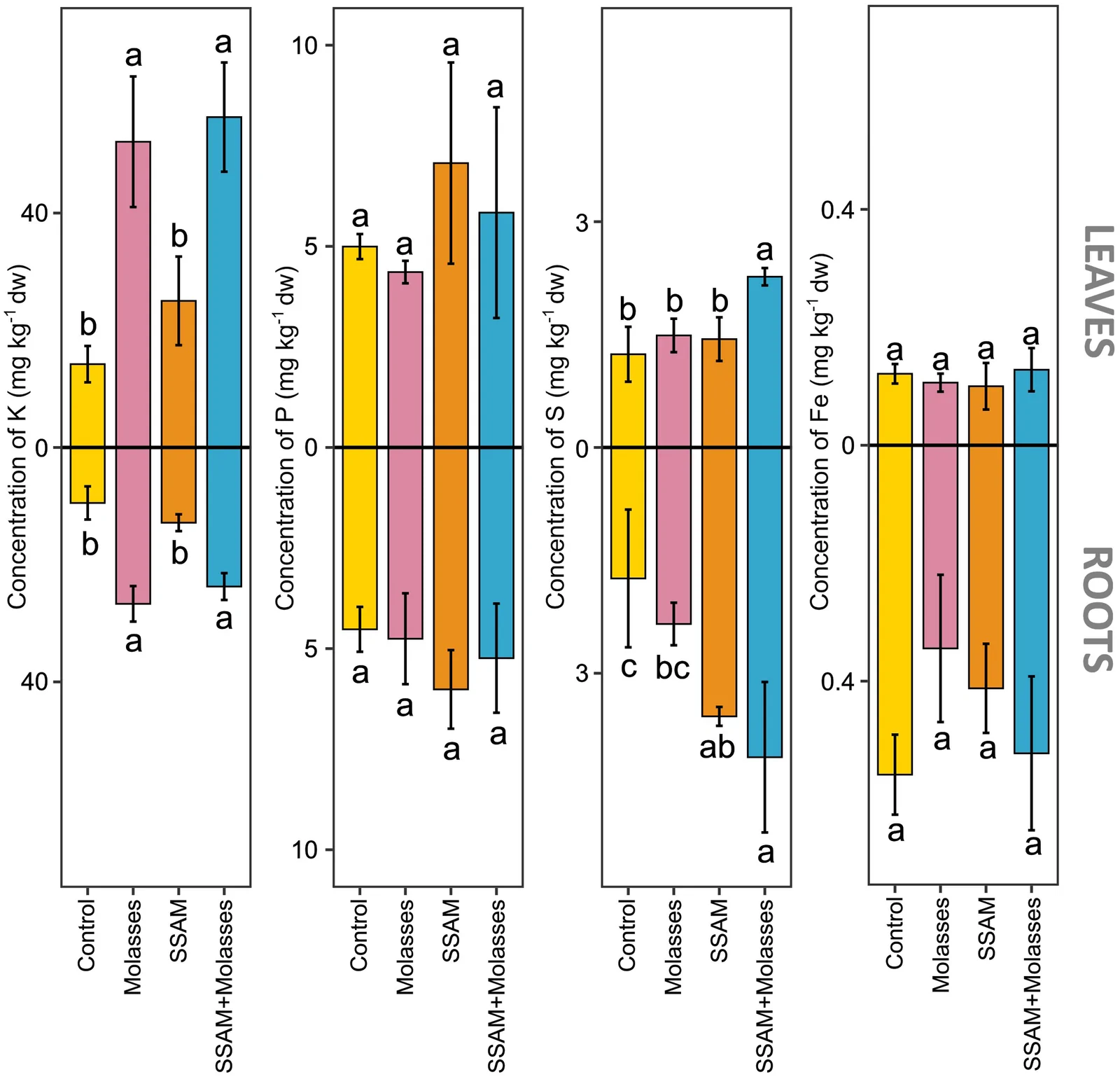
Macro- and micronutrients (from the left: K, P, S, Fe) concentration in basil plants (leaves and roots) under control, molasses, SSAM, and SSAM + molasses treatments. Different letters indicate significant differences according to Tukey’s HSD test (p ≤ 0.05).

The applied treatments affected the elemental composition of basil leaves and roots, although responses varied depending on the nutrient and plant organ analyzed (**Figure 3**). Potassium exhibited one of the most pronounced responses among all analyzed elements. In leaves, both molasses and SSAM+molasses treatments markedly increased K concentrations, reaching levels nearly four times higher than those determined in control plants. A similar pattern was observed in roots, where both molasses-based treatments resulted in approximately a threefold increase in K content compared to the control and SSAM-only treatments. In leaves, P concentrations showed an increasing trend from molasses to SSAM+molasses, with the latter displaying the highest mean values; however, these differences were not statistically meaningful. Similarly, root P concentrations remained comparable among treatments. Sulfur concentration was strongly enhanced by SSAM-containing treatments in both organs. In leaves, the highest S concentration was recorded in the SSAM+molasses treatment, approaching nearly double that of control plants. In roots, S accumulation was likewise substantially elevated under SSAM and SSAM+molasses treatments, with concentrations approximately two- to threefold higher than those in the control and molasses treatment. Two-way factorial analysis showed a significant main effect of molasses on leaf potassium concentration (p < 0.001, η²p = 0.85), whereas the main effect of SSAM (p = 0.097) and the Molasses × SSAM interaction (p = 0.444) were not significant. Root potassium concentration was significantly affected by molasses and showed a significant Molasses × SSAM interaction (p = 0.025, η²p = 0.35). Root sulfur concentration showed a significant main effect of SSAM (p < 0.001, η²p = 0.69), with no significant interaction. Leaf sulfur concentration was significantly affected by both molasses and SSAM and showed a significant Molasses × SSAM interaction (p = 0.049, η²p = 0.29). Neither factor significantly affected phosphorus concentration in leaves or roots (all p > 0.08).

Iron concentrations in leaves remained relatively stable across treatments, indicating that treatments had no pronounced effect on Fe accumulation in aboveground tissues. In roots, however, Fe concentration showed greater variability, with a tendency toward increased levels in the SSAM+molasses and control compared to molasses alone, although overall differences were modest. The factorial model confirms that leaf Fe was unaffected by either factor (molasses p = 0.665; SSAM p = 0.979; interaction p = 0.166). Root Fe, however, showed a significant Molasses × SSAM interaction (F = 9.80, p = 0.009, η²p = 0.45) that the treatment-wise comparison did not resolve. Simple-effects analysis showed that molasses alone significantly decreased root Fe (−0.214, p = 0.013), whereas SSAM significantly increased it when the carrier was present (+0.178, p = 0.032).

### 3.3 MDA content in plants and photosynthetic performance

**Figure 4:**
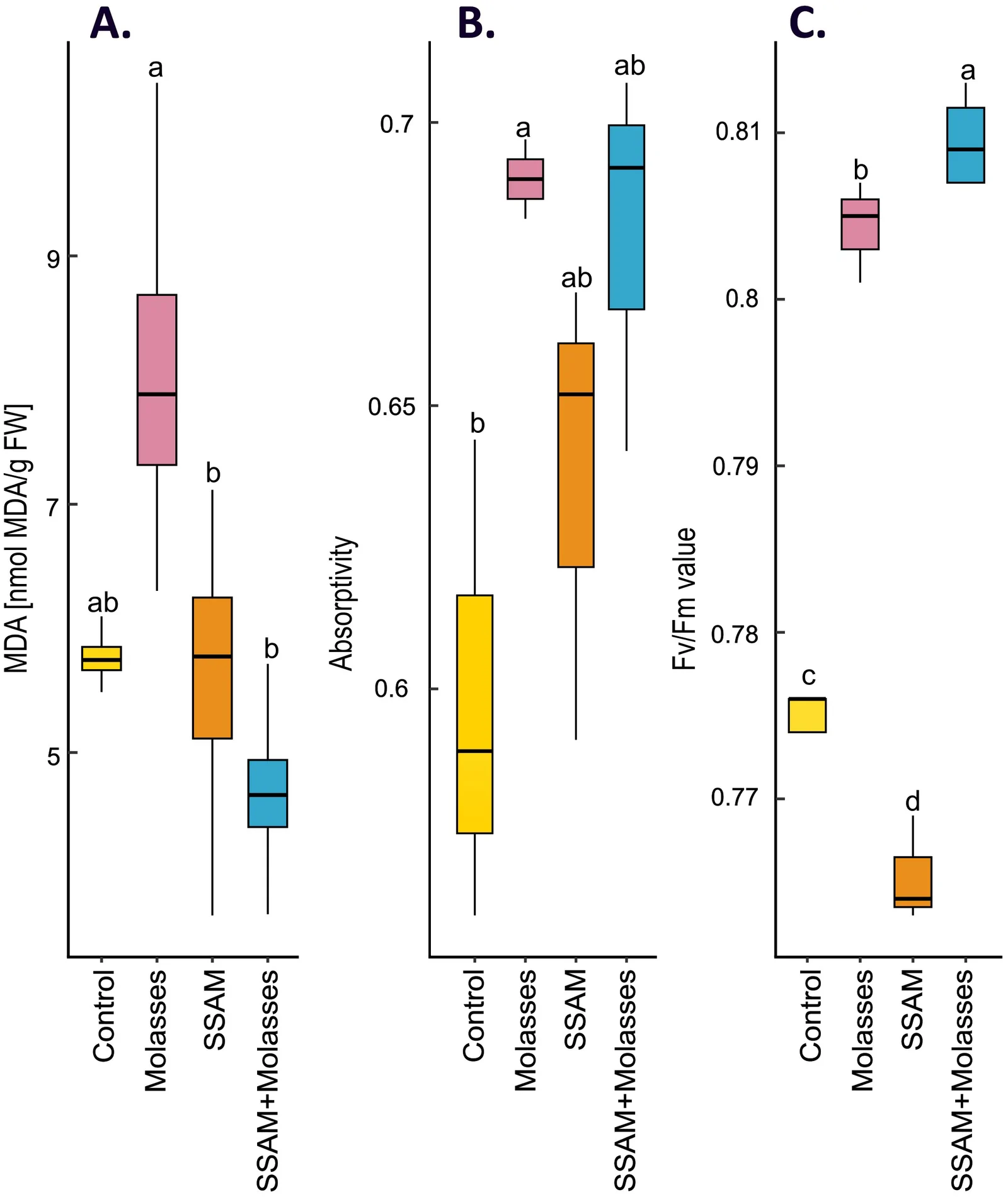
Malondialdehyde (MDA) contents per gram of fresh weight (FW) **(A)**, absorptivity **(B)** and F_V_/F_M_ **(C)**, in basil leaves after 7 weeks of plant growth. Treatments include: Control (untreated), supplementation with molasses, SSAM, SSAM with molasses. Boxes show the interquartile range, horizontal lines indicate medians, and whiskers represent the data range. Different letters indicate significant differences according to Tukey’s HSD test (p ≤ 0.05).

Oxidative stress-related and physiological parameters showed clear, treatment-dependent responses (**Figure 4**). MDA contents varied substantially among treatments. Plants treated with molasses exhibited the highest MDA levels, exceeding those observed in control plants by approximately 40%. Conversely, the SSAM+molasses treatment resulted in markedly lower MDA contents, with values approximately twofold lower than those recorded in molasses-treated plants. Nevertheless, MDA values in plants of the SSAM+molasses treatment were not significantly different from those determined in the control and in SSAM-treated plants.

Leaf absorptivity, a proxy of chlorophyll content, also responded to applied treatments, although the magnitude of change was more moderate. Molasses application resulted in the highest absorptivity values, corresponding to an increase of approximately 15% relative to control plants. SSAM and SSAM+molasses treatments produced intermediate absorptivity values in plants, suggesting a partial enhancement in chlorophyll accumulation relative to the control. More pronounced effects were observed for the maximal efficiency of PSII photochemistry in the dark-adapted state (F_V_/F_M_). The highest F_V_/F_M_ values were recorded in plants treated with SSAM+molasses, followed by molasses alone, both exceeding control levels by approximately 4-5%. In contrast, the SSAM-only treatment resulted in a reduction of F_V_/F_M_ below those of the control.

Two-way factorial analysis revealed significant Molasses × SSAM interactions for MDA (F = 7.47, p = 0.018, η²p = 0.38) and (F_V_/F_M_) (F = 39.14, p < 0.001, η²p = 0.75). Molasses increased MDA relative to the control (+2.35, p = 0.016), while SSAM reduced MDA in the presence of molasses (−3.43, p = 0.002) but had no significant effect when applied alone. SSAM decreased (F_V_/F_M_) in the absence of molasses (−0.0099, p < 0.001) and increased it in its presence (+0.0051, p = 0.008). Absorptivity showed a significant main effect of molasses (p = 0.009, η²p = 0.59), whereas the interaction was not significant (p = 0.247; n = 3).

### 3.4 Soil enzyme activity in response to biofertilizer application

**Figure 5:**
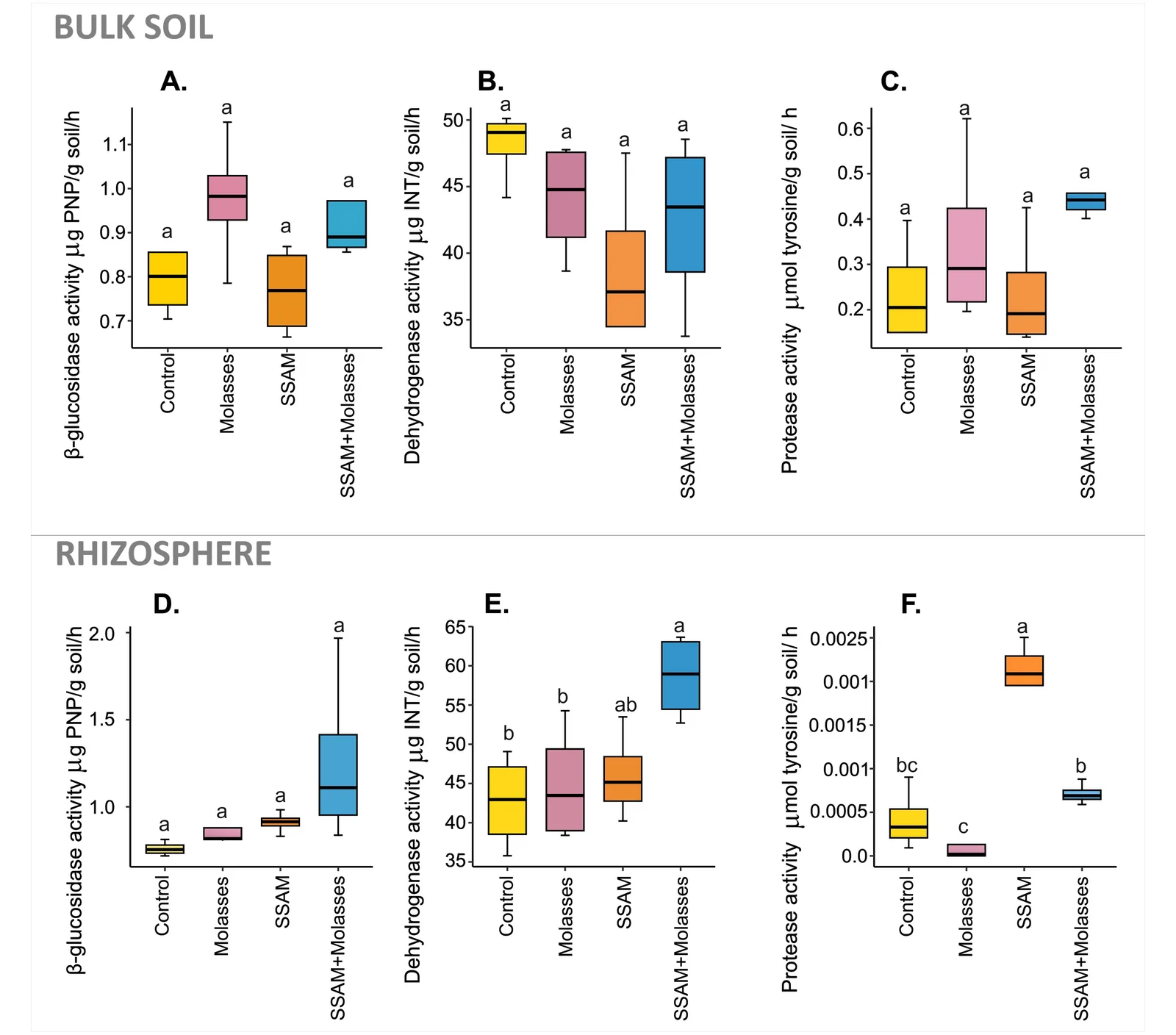
Activities of β-glucosidase **(A, D)**, dehydrogenase **(B, E)**, and protease **(C, F)** in bulk soil **(A–C)** and the basil rhizosphere **(D–F)** under control, molasses, SSAM, and SSAM + molasses treatments. Different letters indicate statistically significant differences between treatments (Tukey’s HSD, p ≤ 0.05).

The activity of three soil enzymes (β-glucosidase, dehydrogenase, and protease) was evaluated in bulk soil and in the rhizosphere under different treatments, revealing distinct response patterns between soil compartments and enzyme types (**Figure 5**). β-glucosidase activity in bulk soil remained relatively stable across all treatments, indicating limited effects on microbial turnover of plant-derived polysaccharides outside the rhizosphere. In contrast, β-glucosidase activity in the rhizosphere showed a clear tendency toward higher values under all treatments compared to the control. The strongest response was observed in the SSAM+molasses treatment, where enzyme activity increased to approximately 1.5 times that of the control. SSAM and molasses alone also stimulated β-glucosidase activity, although the magnitude of these effects was more moderate and variable. Bulk-soil β-glucosidase showed significant main effects of molasses (p = 0.011, η²p = 0.43) and SSAM (p = 0.014, η²p = 0.43), with no significant interaction. Rhizosphere β-glucosidase was not significantly affected in the untransformed model, although the SSAM effect was marginal (p = 0.065) and became significant after log transformation (p = 0.031).

Dehydrogenase activity showed no consistent response to treatments in bulk soil. In the rhizosphere, however, a pronounced treatment effect was observed. The highest dehydrogenase activity occurred in the SSAM+molasses variant, exceeding the control and molasses treatments by approximately 35-40%. Factorially, bulk dehydrogenase responded to a main effect of SSAM only (p = 0.002, η²p = 0.26), while rhizosphere dehydrogenase responded to main effects of both molasses (p = 0.036, η²p = 0.32) and SSAM (p = 0.019, η²p = 0.38) without a significant interaction (p = 0.125, η²p = 0.19).

Protease activity displayed the most distinct and treatment-specific pattern among the analyzed enzymes. In bulk soil, protease activity remained comparable across all treatments, indicating that protein mineralization in the non-rhizosphere fraction was not substantially affected by the applied formulations. In the rhizosphere, however, protease activity was strongly enhanced, particularly in the SSAM treatment. Under this variant, enzyme activity increased by approximately fivefold compared to the control and nearly twentyfold compared to molasses alone. The SSAM+molasses treatment also stimulated protease activity, although to a lesser extent, while molasses alone resulted in the lowest protease activity among all treatments. Rhizosphere protease activity showed a significant Molasses × SSAM interaction (F = 21.10, p < 0.001, η²p = 0.64). SSAM increased protease activity both in the absence (p < 0.001) and presence of molasses (p = 0.005), while molasses reduced protease activity within SSAM-containing treatments (p < 0.001). Bulk-soil protease activity showed significant main effects of molasses (p = 0.037) and SSAM (p = 0.033), with no significant interaction.

### 3.5 Comparison of microbial communities in bulk and rhizosphere soil

**Figure 6:**
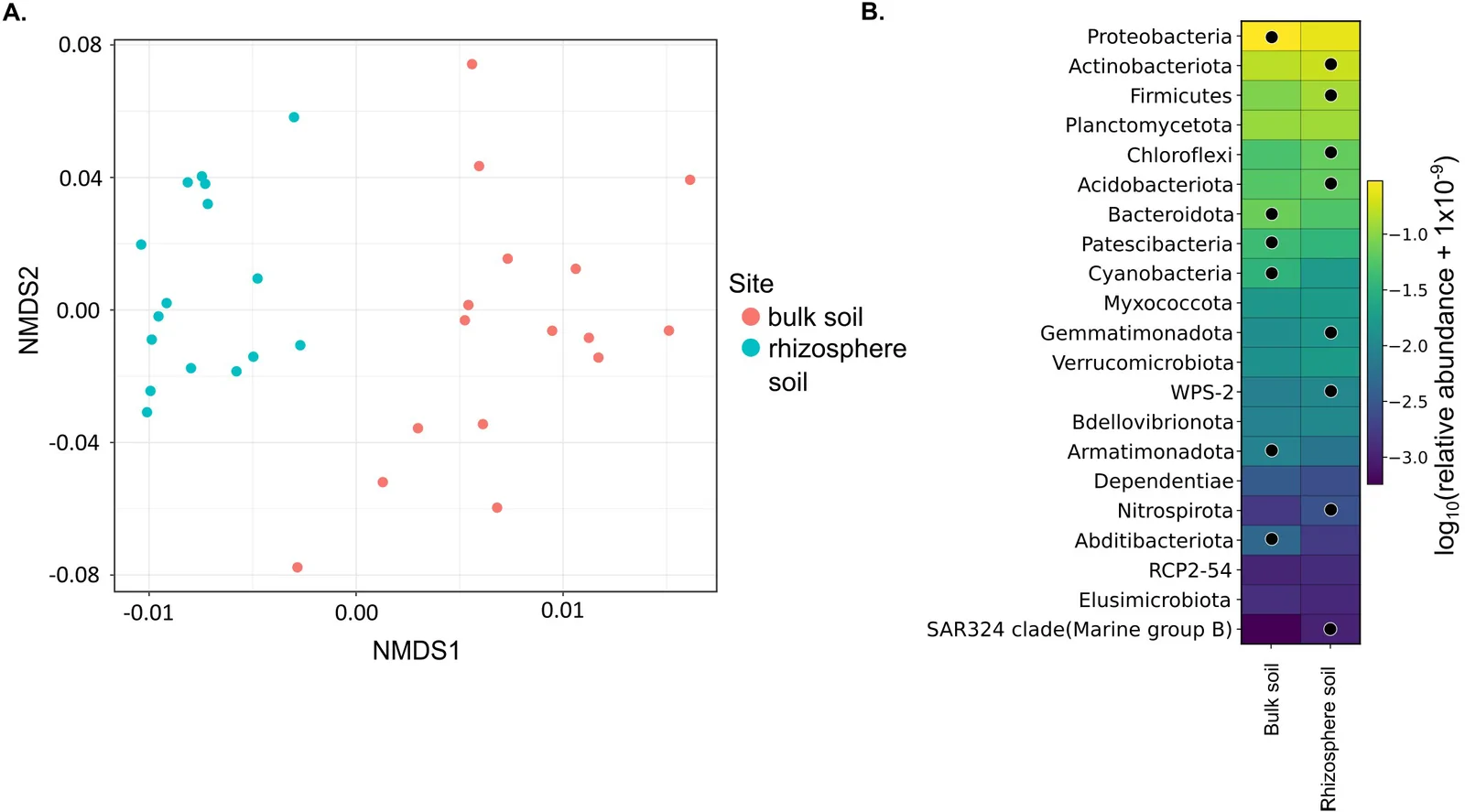
Microbial community in bulk and rhizosphere soil: **(A)** Non-metric multidimensional scaling (NMDS) ordination based on Bray; **(B)** taxonomic composition of bulk and rhizosphere soil at phylum level. Color scale indicates log_10_(relative abundance + 1 × 10^−9^); the pseudocount was added to permit visualization of zero-abundance taxa on a logarithmic scale. The dots indicate an indicator phylum for a given soil compartment.

The composition of microbiomes differed strongly between samples from bulk soil and rhizosphere samples (**Figure 6A**). NMDS analysis showed two distinct clusters based on sample origin, supported with PERMANOVA analysis (R^2^ = 0.4656, p=9.99x10^-5^). These differences were already evident at the phylum level (**Figure 6B**). *Proteobacteria* was the most abundant phylum in both compartments; however, its relative abundance was higher in bulk soil (30.02%) than in the rhizosphere (23.60%). In contrast, the rhizosphere showed greater enrichment of *Actinobacteria* and *Firmicutes*, with relative abundances of 18.22% and 12.86%, respectively, compared to 15.95% and 8.87% in bulk soil. The differences between bulk and rhizosphere soil were further supported by the indicator taxa analysis. In bulk soil, the dominant indicator phyla were *Proteobacteria*, *Bacteroidota*, *Patescibacteria*, and *Cyanobacteria*, whereas the rhizosphere was characterized primarily by *Actinobacteria*, *Firmicutes*, *Chloroflexi*, and *Acidobacteriota*.

### 3.6 Alpha and beta diversity in bulk and rhizosphere soil

**Figure 7:**
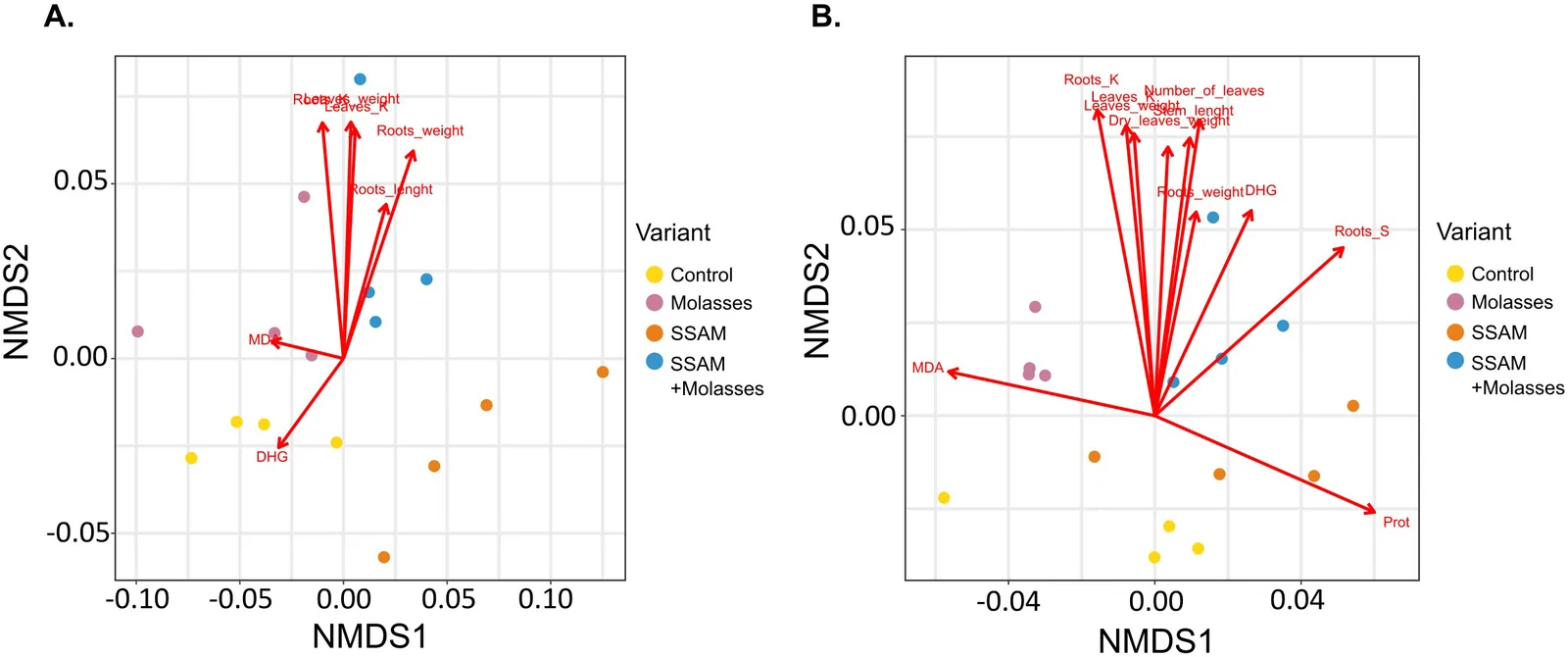
NMDS ordination of microbial communities in bulk soil **(A)** and the rhizosphere **(B)**, based on Bray–Curtis dissimilarities. Red vectors represent significant environmental variables fitted using envfit (p ≤ 0.05).

Alpha diversity analysis did not differ significantly between bulk soil and rhizosphere samples or among treatments. The recorded ranges were 4.224-4.377 for Shannon, 0.975-0.982 for Simpson, and 0.869-0.896 for Pielou index (**Supplementary Table SM.3**). However, the beta-diversity of soil samples was strongly influenced by the applied treatments (**Figure 7**). Differences in community structure among experimental variants were clearly reflected in the NMDS clustering pattern and were further confirmed by PERMANOVA both in bulk soil and the rhizosphere (R^2^ = 0.312; p=1x10–^3^ and R^2^ = 0.335; p=1x10^-3^, respectively). The taxonomic structure was also statistically significantly influenced by Molasses and SSAM addition in bulk soil (R^2^ = 0.117; p=4x10–^3^ and R^2^ = 0.130; p=1x10^-3^, respectively) and rhizosphere soil (R^2^ = 0.117; p=1x10–^3^ and R^2^ = 0.131; p=1x10^-3^, respectively). Significant associations were also observed between soil community composition and plant physiological parameters (e.g., leaves, stems and roots weight, number of leaves, stem length; detailed PERMANOVA results in **Supplementary Table SM.4**). These relationships were further supported by the fitted environmental vectors in the NMDS ordination, which aligned with the SSAM+molasses clusters in both bulk and rhizosphere soil, indicating that this treatment was associated with the strongest shifts in community composition linked to plant growth parameters.

### 3.7 Microbiome community structure and indicator genera in bulk and rhizosphere soil

**Figure 8:**
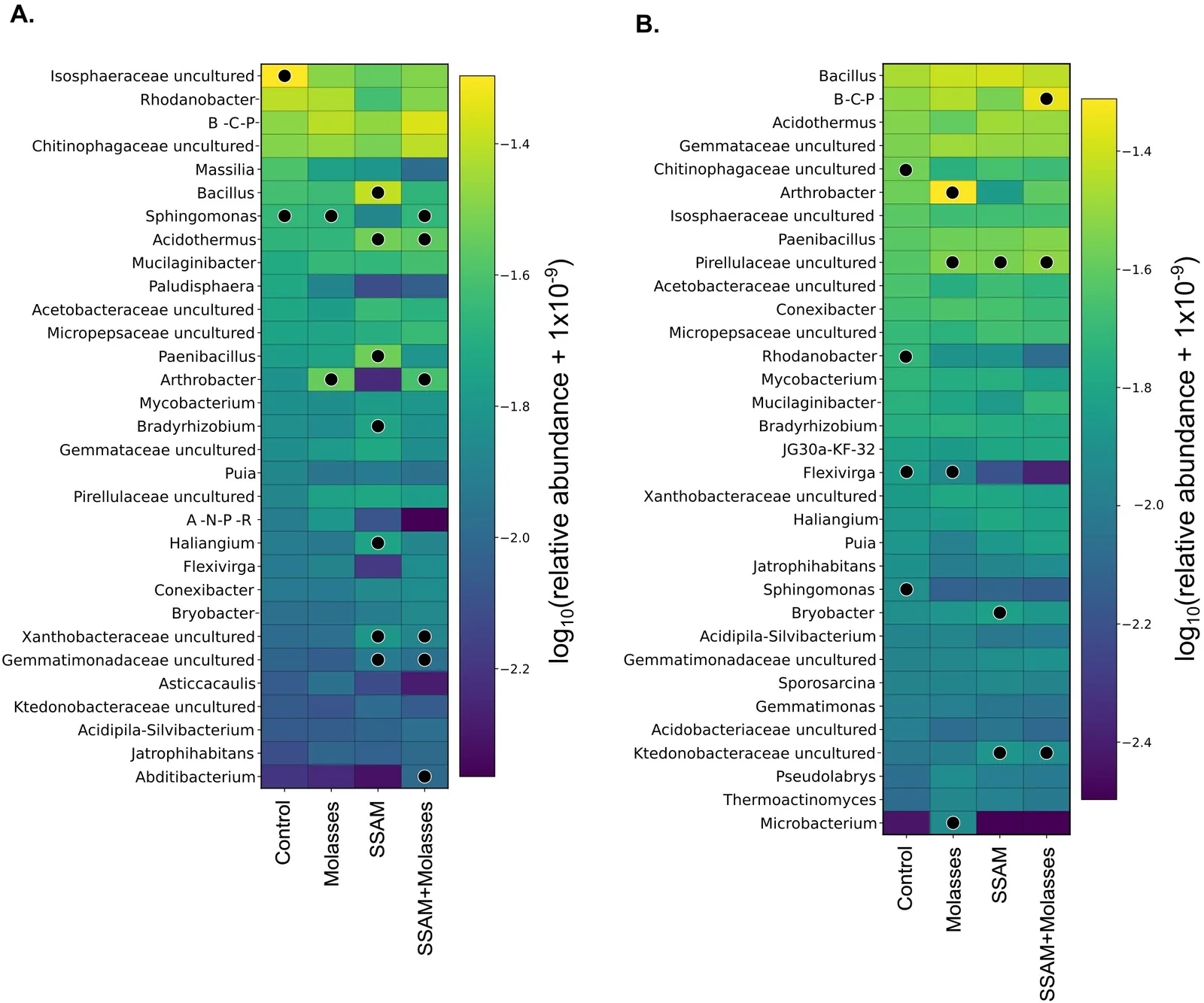
Relative abundances of the dominant genera (>1%) in bulk soil **(A)** and the rhizosphere **(B)**. The color scale represents log_10_(relative abundance + 1 × 10^−9^), and dots indicate indicator genera. B-C-P, Burkholderia–Caballeronia–Paraburkholderia; A-N-P-R, Allorhizobium–Neorhizobium–Pararhizobium–Rhizobium.

At the genus level, treatment-dependent differences in community composition were observed in both bulk and rhizosphere soils, although the dominant taxa varied between soil compartments (**Figure 8**). In bulk soil, the control was dominated by uncultured *Isosphaeraceae* and *Rhodanobacter* (**Figure 8A**). Molasses’ application promoted *Burkholderia-Caballeronia-Paraburkholderia* and maintained high relative abundance of *Rhodanobacter*. The SSAM treatment shifted the community toward increased proportions of *Bacillus* and *Paenibacillus*. In contrast, the combined SSAM+molasses treatment was characterized by the highest abundance of *Burkholderia-Caballeronia-Paraburkholderia* together with *uncultured Chitinophagaceae*. In the rhizosphere soil, treatment effects were also evident, although several dominant genera were shared across treatments (**Figure 8B**). In the control soils, *Bacillus*, *Burkholderia-Caballeronia-Paraburkholderia*, and *Acidothermus*, showed the highest relative abundances. Molasses increased the contribution of *Burkholderia-Caballeronia-Paraburkholderia* while maintaining high levels of *Bacillus*. The SSAM treatment further enhanced *Bacillus* and *Acidothermus*, whereas the combined SSAM+molasses treatment resulted in the highest abundance of *Burkholderia-Caballeronia-Paraburkholderia*, accompanied by consistently high proportions of *Bacillus*.

Indicator species analysis revealed treatment-specific associations in both bulk and rhizosphere soils. In bulk soil, indicator species analysis identified both highly treatment-specific genera and taxa associated with broader treatment groups. Several genera were uniquely linked to a single treatment. Uncultured *Isosphaeraceae* was exclusively associated with the control, whereas *Abditibacterium* was specific to the SSAM+molasses treatment. The greatest number of unique indicator genera was observed for SSAM, with *Bacillus* and *Paenibacillus* being the most prominent. In contrast, some genera were associated with multiple treatments, suggesting broader ecological tolerance or shared environmental drivers. *Sphingomonas* was identified as an indicator for both the control and molasses treatments. *Acidothermus*, uncultured *Xanthobacteraceae*, and uncultured *Gemmatimonadaceae* were associated with both treatments containing SSAM. *Arthrobacter* was identified as an indicator of treatments that included molasses.

## 4 Discussion

### 4.1 Biofertilizer-driven enhancement of plant growth, nutrient uptake and lipid peroxidation

The results obtained in this study indicate that the effects of the combined formulation arose from two distinct contributions of beet molasses: its role as a readily available carbon source and its direct contribution of nutrients, particularly K. The factorial analysis allowed these effects to be distinguished from those associated with SSAM. Leaf potassium showed a strong response to molasses, whereas neither the main effect of SSAM nor the Molasses × SSAM interaction was significant, indicating that potassium accumulation was primarily driven by direct nutrient supply from the carrier. A similar molasses-dominated and largely additive response was observed for leaf biomass, leaf number, stem length, and absorptivity. By contrast, MDA, F_V_/F_M_, root iron concentration, and rhizosphere protease activity showed significant Molasses × SSAM interactions, indicating that the biological response to SSAM was contingent on the presence of molasses. Specifically, SSAM reduced MDA and increased F_V_/F_M_ only when applied with molasses, while its effects on root Fe and rhizosphere protease activity also differed between carrier levels. Similar carrier-dependent responses have been reported for plant-beneficial rhizobacteria and for formulated bacterial cell-free supernatants (Tahir et al., 2022; Gonzalez-Montfort et al., 2022). These findings support the broader conclusion that formulation components can modify the biological outcome of microbial inputs. Nevertheless, our results do not demonstrate that molasses directly enhanced the intrinsic activity or stability of SSAM. Therefore, the effects of the combined formulation should not be interpreted as a uniform synergistic response across all measured parameters. Rather, improvements in biomass production and K accumulation appear to be attributable mainly to molasses acting as a nutrient and carbon source, whereas SSAM contributed primarily as a potentiating factor affecting physiological quality, including redox balance, photochemical efficiency, root-level iron handling, and rhizosphere enzymatic activity. Relative to previous studies on basil, the magnitude of the growth responses observed here was substantial under controlled experimental conditions. In hydroponic systems, bacterial fertilization increased total basil yield by 18.94% compared with a 50% mineral-fertilizer control (Dasgan et al., 2022). Under field conditions, inoculation of basil with arbuscular mycorrhizal fungi (AMF) and plant growth promoting rhizobacteria (PGPR) significantly increased plant height (+27%), root length (+43%), and biomass (increase of root FW by 113%) compared to the unamended control (Khalediyan et al., 2021). Although differences in cultivation system, formulation type, experimental duration, and measured endpoints preclude direct quantitative comparisons, these studies provide context for the relatively large growth response observed in the present pot experiment. Field trials are nevertheless required to determine whether this response is maintained under agronomic conditions.

The nutrient-uptake pattern further clarifies the complementary roles of the two components in the formulation. The near-fourfold increase in leaf K in both molasses-containing treatments directly reflects the high K content of molasses (Otani et al., 2023; Palmonari et al., 2020) and likely contributed to the stimulation of leaf development through improved turgor maintenance, stomatal regulation, and photosynthetic support (Pettigrew, 2008; Kumar et al., 2020). By contrast, the S response was more closely associated with SSAM than with molasses, as SSAM-containing treatments showed consistently elevated S concentrations, particularly in roots. This suggests that the bacterial metabolite fraction influenced S acquisition and/or assimilation and may have increased demand for S-containing compounds involved in stress protection, redox regulation and energy transfer in photosynthesis (Aziz et al., 2016; Capaldi et al., 2015; Mukherjee et al., 2025). In contrast, phosphorus remained largely unaffected, suggesting that P availability was not a limiting factor in our study or that the treatments did not significantly alter its mobility.

The patterns of Fe uptake add a further layer of complexity. Leaf Fe concentration was unaffected by either component and showed no significant interaction. However, its stability under a treatment producing markedly greater leaf biomass indicates that Fe acquisition kept pace with growth and that no dilution-related deficiency developed. This should be interpreted as evidence of Fe sufficiency rather than siderophore-mediated Fe gain and is consistent with tight homeostatic regulation of Fe translocation to the shoot (Connorton et al., 2017). Root Fe concentration showed a significant Molasses × SSAM interaction. Molasses alone reduced root Fe, whereas SSAM restored it in the presence of the carrier, returning concentrations to approximately the control level. Previous studies suggest that labile rhizosphere carbon can stimulate microbial resource demand, including competition with plants for Fe, whereas bacterial siderophores may facilitate Fe acquisition by maintaining it in chelated forms (Bar-Ness et al., 1992; Fan et al., 2022; Radzki et al., 2013; Gao et al., 2022; Lozano-González et al., 2025). These findings support a working hypothesis that molasses increased microbial Fe demand while SSAM helped maintain Fe availability at the root–soil interface. However, because Fe speciation, microbial immobilization, siderophore–Fe complex formation, and root Fe-uptake responses were not measured, the interaction demonstrates a molasses-dependent effect of SSAM but does not establish the underlying mechanism. Metabolite-mediated signaling, microbially mediated redox regulation, and broader nutrient-cycling effects are equally plausible, with the strong SSAM effect on root S and the Molasses × SSAM interaction for leaf S providing a concrete example of the established coupling between S and Fe metabolism in plants (Astolfi et al., 2021). Despite the lack of change in leaf Fe concentration following metabolite treatments the photosynthetic capacity improvement of plants supplemented with SSAM+molasses could be observed. This is supported by the increased leaf absorptivity and F_V_/F_M_ values, indicating enhanced chlorophyll content and more efficient PSII photochemistry. Such an effect was likely due to greater nutrient accumulation, as the high K supply provided by molasses may have contributed to enhanced stomatal regulation and photosynthetic performance (Pettigrew, 2008). Absorptivity indeed responded to molasses alone, consistent with such a nutritional route. The F_V_/F_M_ response, however, was interactive rather than additive: SSAM lowered photochemical efficiency when applied on its own, yet raised it above all other treatments when combined with the carrier. The metabolite fraction is therefore not beneficial to PSII photochemistry in itself. Its advantage is expressed only within a carbon-enriched rhizosphere, and cannot be explained by K supply alone. With respect to oxidative stress, molasses-treated plants exhibited MDA concentrations approximately 40% above controls, the highest of all variants, indicating an oxidative cost on plant metabolism despite growth stimulation, most likely through intensified microbial respiration and associated redox disturbances in the root environment (Gill and Tuteja, 2010; Kuzyakov and Blagodatskaya, 2015). SSAM reduced MDA only when applied together with molasses, whereas it had no measurable effect when applied alone and molasses alone increased MDA relative to the control. Thus, the demonstrated effect is a conditional reduction in MDA under the combined treatment. The reduction in MDA indicates lower lipid peroxidation and suggests that the combined treatment alleviated oxidative damage. However, the physiological underlying mechanism remains unresolved. One possibility is that siderophore-mediated changes in Fe availability could potentially influence antioxidant enzymes such as catalase, superoxide dismutase and peroxidase (Sharma and Johri, 2003), but leaf Fe concentrations were not significantly affected by the treatments and antioxidant enzyme activities were not measured. Alternatively, the increased S concentrations associated with SSAM may have contributed to antioxidant protection, as S is required for glutathione biosynthesis and is closely linked to cellular redox metabolism (Maruyama-Nakashita and Ohkama-Ohtsu, 2017). However, neither mechanism can be confirmed from the present data. Distinguishing among enhanced enzymatic antioxidant activity, increased non-enzymatic scavenging capacity, and reduced production of reactive oxygen species will require direct measurements of antioxidant enzymes, glutathione-related metabolites, and Fe speciation in the rhizosphere (Song et al., 2024).

### 4.2 Effects of biofertilizer on soil properties and microbial functioning

Enzymatic and microbiological responses were consistently compartment-specific: all statistically significant effects were detected in the rhizosphere, whereas bulk soil remained comparatively stable across treatments. This pattern is consistent with the formulations acting primarily at the soil–root interface rather than through generalized enrichment of the whole substrate, suggesting that their effects were mediated by rhizosphere processes. In this zone, root exudates provide localized carbon and signaling inputs that selectively stimulate microbial activity and contribute to the assembly of root-associated microbial communities (Kuzyakov and Blagodatskaya, 2015; Raaijmakers et al., 2009; Sasse et al., 2018). Importantly, each treatment generated a distinct rhizosphere profile corresponding to a different plant treatment. The strongest plant response under SSAM+molasses coincided with the most integrated rhizosphere response, characterized by highest β-glucosidase and dehydrogenase activities, elevated protease activity and a pronounced microbiome shift.

The enzyme data coincide with a functional interpretation of this hierarchy. β-glucosidase and dehydrogenase activities peaked under the SSAM+molasses treatment, suggesting that the combined formulation most effectively translated carbon inputs into elevated microbial metabolic intensity. Dehydrogenase showed a stronger treatment hierarchy, with highest activity in SSAM+molasses, intermediate activity in SSAM, and values near the control in molasses alone. This agrees with observations in the maize rhizosphere, where molasses increased β-glucosidase but did not significantly stimulate dehydrogenase (Nugroho et al., 2023). Molasses alone provided a substrate for microbial activation but did not achieve comparable overall activity, indicating that SSAM improved the functional organization of the rhizosphere rather than simply supplying additional carbon. This interpretation aligns with field studies in spinach and tomato, where the strongest growth responses occurred when bacterial inoculants were combined with organic amendments (Song et al., 2015). Protease activity followed an inverse pattern, peaking in SSAM alone, intermediate in SSAM+molasses, and lowest under molasses. This suggests that SSAM stimulated a more specialized, proteolytic mode of rhizosphere functioning. The reduction of protease activity by molasses alone is consistent with the concept that labile C can repress protease synthesis, because protease partly functions as a C-acquiring enzyme when readily available sugars are limited (Geisseler and Horwath, 2008). Therefore, high protease activity under SSAM alone, together with only moderate plant growth, may reflect an imbalance between N-acquiring and C-cycling processes, potentially reducing microbial C-use efficiency (Sinsabaugh et al., 2016). In contrast, SSAM+molasses appears to promote a more balanced rhizosphere, integrating protein turnover with intensified C cycling and more efficient nutrient mobilization.

Alpha diversity remained stable across all treatments and compartments, which might be associated with the reorganization of community composition by the formulations rather than reducing richness. Instead, the main response was compositional: beta diversity differed significantly between treatments in both bulk soil and the rhizosphere, and community structure was associated with plant growth traits, with ordination vectors pointing toward SSAM+molasses clusters. This agrees with studies showing that microbial inoculants and biofertilizers often restructure community composition without changing richness, and that community structure rather than alpha diversity better explains treatment-dependent soil functionality and plant performance (Li et al., 2024; Zhou et al., 2020). Therefore, the superior plant response under SSAM+molasses likely co-occurred with a rhizosphere assemblage compositionally consistent with favorable plant traits, although the causal direction of this association cannot be resolved by the present design. At the taxonomic level, these treatment-specific shifts broadly aligned with the functional ecology of the dominant groups, although their functions were not directly measured. Molasses was associated with greater relative abundance of enrichment favored *Burkholderia-Caballeronia-Paraburkholderia* (BCP) while maintaining high *Bacillus* abundance. BCP members are typical copiotrophs within β-Proteobacteria, whose enrichment under high-carbon inputs is well established (Fierer et al., 2007), and their presence in the rhizosphere has been associated with a range of plant-beneficial traits including N fixation, phosphate solubilization, and phytohormone production (Park et al., 2025; Rojas-Rojas et al., 2025). SSAM was associated with increased proportions of Bacillus, Paenibacillus, and Acidothermus. Bacillus is widely recognized for rhizosphere competence and hydrolytic versatility, whereas Paenibacillus includes taxa associated with nitrogen fixation and complex-substrate degradation (Mahapatra et al., 2022; Langendries and Goormachtig, 2021; Lal and Tabacchioni, 2009). The ecological significance of Acidothermus, uncultured Chitinophagaceae, and Abditibacterium in the present system remains uncertain. Therefore, these taxonomic shifts should not be interpreted as direct evidence of specific microbial functions or mechanisms underlying plant growth. The combined SSAM+molasses treatment produced the highest BCP abundance while retaining elevated *Bacillus*, effectively integrating both microbial signatures. The ecological significance of Acidothermus, uncultured Chitinophagaceae, and Abditibacterium in the present system remains uncertain. Overall, the taxonomic patterns were consistent with molasses favoring carbon-responsive taxa and SSAM being associated with taxa possessing hydrolytic potential. Nevertheless, these functional interpretations are based on previously reported characteristics of related taxa and should not be taken as direct evidence that the observed community shifts mediated the plant growth response.

The interpretation of results is constrained by the small number of biological observations. Although four biological replicates per treatment allowed the detection of strong treatment-associated differences, this level of replication limits the statistical power to characterize within-treatment microbiome heterogeneity, robustly estimate biological dispersion, and detect smaller treatment effects. Consequently, the observed relationships among microbiome composition, rhizosphere enzyme activity, and plant performance should be interpreted as co-occurring treatment responses rather than as a demonstrated causal chain.

Validation in larger greenhouse experiments with increased replication and independent experimental runs, followed by field trials across different soils and environmental conditions, will be required to determine the robustness and generalizability of these patterns. Establishing mechanistic links will additionally require time-resolved functional approaches, including metatranscriptomics, metaproteomics, and metabolomics, to identify the microbial genes, proteins, and metabolites actively associated with plant responses (Kumar et al., 2023). These analyses should be combined with manipulative experiments using synthetic microbial communities, defined re-inoculation, or selective microbial suppression. Such approaches would help determine whether microbiome restructuring contributes directly to plant performance, occurs as a consequence of altered plant physiology, or reflects reciprocal plant–microbiome responses.

### 4.3 Mechanistic insights and implications for sustainable biofertilizer development

The overall hierarchy of plant responses observed in this was accompanied by distinct treatment-associated rhizosphere profiles. Molasses alone stimulated plant growth and was associated with a rhizosphere predominantly shaped by carbon enrichment. SSAM alone coincided with a more selective, strongly proteolytic rhizosphere response, yet lacked the broader metabolic activation required to support robust plant development. By contrast, SSAM+molasses treatment combined intensified carbon turnover, high overall microbial activity, and a distinct plant-associated microbiome structure with the highest growth, lower MDA, and higher F_V_/F_M_. These parallel responses are consistent with a potential contribution of rhizosphere restructuring to plant performance, but they do not demonstrate that the microbiome changes caused the observed physiological effects.

Taken together, the results support a testable working model in which the response to the SSAM+molasses formulation may arise from complementary processes at the plant–soil interface rather than from a single dominant factor. Molasses functioned primarily as an energy- and nutrient-rich component associated with increased plant growth, rhizosphere carbon turnover, and potassium supply, but also with elevated MDA. SSAM had little effect on biomass when applied alone, whereas its application with molasses was associated with lower lipid peroxidation, higher photochemical efficiency, altered and a distinct rhizosphere enzymatic and taxonomic profile. An important constraint of the present study is that the relationship between rhizosphere reorganization and plant performance is correlational rather than causal. Establishing causality would require manipulative approaches such as soil sterilization with defined re-inoculation, selective microbial suppression, or time-resolved sampling that resolves the temporal ordering of microbial and plant responses. The present findings therefore generate specific mechanistic hypotheses rather than establishing microbiome-mediated plant growth promotion.

From an applied perspective, these findings support further development of biofertilizers based on defined microbial metabolites and sustainable organic carriers (Gahlot, 2026). Compared with live inoculants, such products may offer greater control over composition, reduced dependence on microbial survival after application, and greater formulation flexibility (Malusá and Vassilev, 2014). However, the present study should be regarded as a proof of concept rather than evidence of commercial or field-scale readiness. Further development will require evaluation of formulation stability, shelf life, metabolite activity, microbiological purity, packaging compatibility, and batch-to-batch consistency. Production-scale studies should also address fermentation efficiency, standardization of SSAM and molasses, downstream processing, quality control, and the costs of production, storage, transport, and application. Commercialization will depend on regulatory classification and demonstration of product identity, safety, stability, efficacy, and manufacturing consistency. Finally, larger greenhouse experiments and multi-site field trials will be needed to determine effective application rates, crop and soil specificity, environmental safety, and agronomic performance under variable conditions.

The novelty of the proposed solution lies in several interconnected aspects. First, the active component was not a live inoculant, but a cell-free metabolite fraction containing SSAM, which broadens the concept of biofertilization beyond microbial survival and colonization. Second, the formulation combined this metabolite fraction with molasses as a low-cost organic carrier, creating a system in which the carrier was not inert, but functionally involved in shaping plant and microbial responses. Finally, the study demonstrates that such a formulation can simultaneously improve plant growth, alleviate oxidative stress, and restructure the rhizosphere microbiome, which is still rarely documented within one integrated experimental framework.

## Data Availability

The original contributions presented in the study are included in the article/**Supplementary Material**. Further inquiries can be directed to the corresponding authors.
