# Supplementary material for: Molasses-amended siderophore biofertilizer reshapes the rhizosphere microbiome, promotes growth and reduces lipid peroxidation in Ocimum basilicum L
Source: Front Plant Sci. 2026 Aug 13;17:1879696. doi: 10.3389/fpls.2026.1879696 (PMC13518571; doi:10.3389/fpls.2026.1879696)
Supplement: Supplementary file 1 [file DataSheet1.docx]

Table SM.1.Two-way factorial ANOVA (Type III sums of squares). p-values and partial eta-squared (η²p) for main effects of molasses (Mol.) and SSAM, and their interaction (M×S), n=4

| **Parameter** | **Mol. p** | **Mol. η²p** | **SSAM p** | **SSAM η²p** | **M×S p** | **M×S η²p** |
| --- | --- | --- | --- | --- | --- | --- |
| Dry leaf weight | <0.001 | 0.818 | 0.022 | 0.365 | 0.397 | 0.061 |
| Dry stem weight | <0.001 | 0.847 | 0.187 | 0.140 | 0.030 | 0.335 |
| Dry root weight | 0.029 | 0.340 | 0.374 | 0.066 | 0.108 | 0.201 |
| Number of leaves | <0.001 | 0.824 | 0.003 | 0.535 | 0.200 | 0.133 |
| Stem length | <0.001 | 0.633 | 0.178 | 0.146 | 0.723 | 0.011 |
| Root length | 0.270 | 0.100 | 0.105 | 0.204 | 0.040 | 0.306 |
| Leaves K | <0.001 | 0.852 | 0.097 | 0.213 | 0.444 | 0.050 |
| Leaves Fe | 0.665 | 0.016 | 0.979 | <0.001 | 0.166 | 0.153 |
| Leaves P | 0.327 | 0.080 | 0.075 | 0.240 | 0.748 | 0.009 |
| Leaves S | 0.002 | 0.581 | 0.003 | 0.534 | 0.049 | 0.285 |
| Roots K | <0.001 | 0.915 | 0.868 | 0.002 | 0.025 | 0.352 |
| Roots Fe | 0.336 | 0.077 | 0.766 | 0.008 | 0.009 | 0.450 |
| Roots P | 0.614 | 0.022 | 0.084 | 0.229 | 0.354 | 0.072 |
| Roots S | 0.125 | 0.185 | <0.001 | 0.691 | 0.930 | 0.001 |
| β-glucosidase (bulk) | 0.011 | 0.431 | 0.688 | 0.014 | 0.980 | <0.001 |
| β-glucosidase (rhizo.) | 0.105 | 0.204 | 0.065 | 0.257 | 0.396 | 0.061 |
| Dehydrogenase (bulk) | 0.875 | 0.002 | 0.064 | 0.258 | 0.187 | 0.140 |
| Dehydrogenase (rhizo.) | 0.036 | 0.317 | 0.019 | 0.380 | 0.125 | 0.185 |
| Protease (bulk) | 0.037 | 0.315 | 0.537 | 0.033 | 0.519 | 0.036 |
| Protease (rhizo.) | <0.001 | 0.804 | <0.001 | 0.881 | <0.001 | 0.637 |
| MDA | 0.249 | 0.109 | 0.010 | 0.435 | 0.018 | 0.384 |
| Fv/Fm | <0.001 | 0.986 | 0.068 | 0.234 | <0.001 | 0.751 |
| Absorptivity | 0.009 | 0.590 | 0.467 | 0.068 | 0.247 | 0.163 |

Table SM.2. Simple effects of SSAM and molasses for response variables showing significant factorial interactions.

| **Parameter** | **Effect of** | **At level** | **Difference** | **t** | **p** | **Sig.** |
| --- | --- | --- | --- | --- | --- | --- |
| Fv/Fm | Sidero | Molasses = No | **-0.0099** | -5.60 | 0.0001 | ******* |
| Fv/Fm | Sidero | Molasses = Yes | **+0.0051** | 3.15 | 0.0077 | ****** |
| Fv/Fm | Molasses | SSAM = No | **+0.0292** | 19.14 | <0.0001 | ******* |
| Fv/Fm | Molasses | SSAM = Yes | **+0.0442** | 23.97 | <0.0001 | ******* |
| MDA | Sidero | Molasses = No | -0.181 | -0.22 | 0.8333 | ns |
| MDA | Sidero | Molasses = Yes | **-3.435** | -4.08 | 0.0015 | ****** |
| MDA | Molasses | SSAM = No | **+2.348** | 2.79 | 0.0164 | ***** |
| MDA | Molasses | SSAM = Yes | -0.906 | -1.08 | 0.3031 | ns |
| Protease_rhizo | Sidero | Molasses = No | **+1.74e-3** | 9.90 | <0.0001 | ******* |
| Protease_rhizo | Sidero | Molasses = Yes | **+6.00e-4** | 3.40 | 0.0052 | ****** |
| Protease_rhizo | Molasses | SSAM = No | -3.02e-4 | -1.71 | 0.1123 | ns |
| Protease_rhizo | Molasses | SSAM = Yes | **-1.45e-3** | -8.21 | <0.0001 | ******* |
| Roots_Fe | Sidero | Molasses = No | -0.146 | -2.00 | 0.0689 | ns |
| Roots_Fe | Sidero | Molasses = Yes | **+0.178** | 2.43 | 0.0318 | ***** |
| Roots_Fe | Molasses | SSAM = No | **-0.214** | -2.92 | 0.0128 | ***** |
| Roots_Fe | Molasses | SSAM = Yes | +0.110 | 1.50 | 0.1583 | ns |
| Dry_stem_weight | Sidero | Molasses = No | -0.030 | -0.75 | 0.4692 | ns |
| Dry_stem_weight | Sidero | Molasses = Yes | **+0.110** | 2.73 | 0.0184 | ***** |
| Dry_stem_weight | Molasses | SSAM = No | **+0.163** | 4.03 | 0.0017 | ****** |
| Dry_stem_weight | Molasses | SSAM = Yes | **+0.304** | 7.50 | <0.0001 | ******* |
| Root_length | Sidero | Molasses = No | -0.475 | -0.38 | 0.7081 | ns |
| Root_length | Sidero | Molasses = Yes | **+3.550** | 2.87 | 0.0142 | ***** |
| Root_length | Molasses | SSAM = No | -1.000 | -0.81 | 0.4353 | ns |
| Root_length | Molasses | SSAM = Yes | **+3.025** | 2.44 | 0.0311 | ***** |
| Leaves_S | Sidero | Molasses = No | +0.201 | 1.07 | 0.3037 | ns |
| Leaves_S | Sidero | Molasses = Yes | **+0.780** | 4.17 | 0.0013 | ****** |
| Leaves_S | Molasses | SSAM = No | +0.250 | 1.34 | 0.2063 | ns |
| Leaves_S | Molasses | SSAM = Yes | **+0.829** | 4.43 | 0.0008 | ******* |
| Roots_K | Sidero | Molasses = No | +3.368 | 1.93 | 0.0781 | ns |
| Roots_K | Sidero | Molasses = Yes | -2.950 | -1.69 | 0.1175 | ns |
| Roots_K | Molasses | SSAM = No | **+17.21** | 9.84 | <0.0001 | ******* |
| Roots_K | Molasses | SSAM = Yes | **+10.89** | 6.23 | <0.0001 | ******* |

Table SM.3. The alpha diversity indices (Shannon, Simpson, and Pielou’s evenness) from the microbiome in soil sampled at the end of the experiment in soil bulk and rhizosphere soils. Treatments include: Control (no supplementation), supplementation with SSAM, molasses, and SSAM with molasses.

|  | Bulk | | | Rhizosphere | | |
| --- | --- | --- | --- | --- | --- | --- |
|  | Shannon | Simpson | Pielou | Shannon | Simpson | Pielou |
| Control | 4,334263 | 0,980504 | 0,887659 | 4,320849 | 0,980156 | 0,884912 |
| Molasses | 4,320478 | 0,980233 | 0,884836 | 4,313053 | 0,979516 | 0,883315 |
| SSAM | 4,356898 | 0,981556 | 0,892295 | 4,342127 | 0,981024 | 0,889270 |
| SSAM+molasses | 4,321596 | 0,980337 | 0,885065 | 4,313069 | 0,980211 | 0,883318 |

Table SM.4. Results of separate PERMANOVA analyses evaluating the relationship between environmental and plant-growth variables and bacterial community structure in bulk and rhizosphere soil.

|  | Bulk | | Rhizosphere | |
| --- | --- | --- | --- | --- |
| Variable | R2 | p_value | R2 | p_value |
| Variant | 0.31203998 | 0.001 | 0.33512541 | 0.001 |
| Molasses | 0.11718435 | 0.004 | 0.11651323 | 0.001 |
| SSAM | 0.13037560 | 0.001 | 0.13108403 | 0.001 |
| Leaves weight | 0.13037560 | 0.001 | 0.11933797 | 0.002 |
| Stem weight | 0.10436566 | 0.008 | 0.11969309 | 0.002 |
| Roots weight | 0.10208398 | 0.016 | 0.11221198 | 0.003 |
| Dry leaves weight | 0.08759550 | 0.068 | 0.11812721 | 0.002 |
| Dry stem weight | 0.10017833 | 0.020 | 0.08425483 | 0.081 |
| Dry roots weight | 0.08814460 | 0.066 | 0.09007555 | 0.052 |
| Number of leaves | 0.08076700 | 0.145 | 0.12468335 | 0.001 |
| Stem lenght | 0.09447686 | 0.029 | 0.11082546 | 0.001 |
| Roots lenght | 0.09429313 | 0.025 | 0.09890895 | 0.014 |
| Leaves Mg | 0.07013134 | 0.309 | 0.10354995 | 0.011 |
| Leaves K | 0.11533664 | 0.007 | 0.10822594 | 0.005 |
| Leaves Ca | 0.10168091 | 0.017 | 0.10091545 | 0.013 |
| Leaves Fe | 0.12309228 | 0.002 | 0.06616192 | 0.482 |
| Leaves P | 0.06319943 | 0.557 | 0.08432160 | 0.083 |
| Leaves S | 0.07666206 | 0.179 | 0.10853498 | 0.006 |
| Roots Mg | 0.08480400 | 0.067 | 0.08742003 | 0.059 |
| Roots K | 0.12027058 | 0.002 | 0.10630241 | 0.009 |
| Roots Ca | 0.10015300 | 0.026 | 0.06233163 | 0.577 |
| Roots Fe | 0.07273063 | 0.262 | 0.08116475 | 0.124 |
| Roots P | 0.06919146 | 0.321 | 0.07681235 | 0.168 |
| Roots S | 0.08796775 | 0.065 | 0.11535641 | 0.003 |
| MDA | 0.09731908 | 0.022 | 0.09054599 | 0.037 |
| B-Glucosidases | 0.06458935 | 0.523 | 0.10133703 | 0.003 |
| Dehydrogenases | 0.10397395 | 0.012 | 0.09357008 | 0.032 |
| Proteases | 0.07597493 | 0.181 | 0.10044577 | 0.013 |

Supplementary Figure S1.

*Figure 1. Morphology of control (untreated) plants (A), plants treated with molasses (B), SSAM (C) and SSAM + molasses (D). Images were taken after 7 weeks of plant growth under controlled conditions. Scale bars correspond to 4 cm (n = 4).*

Based on qualitative visual assessment of the photographic data, plants supplemented with SSAM+molasses (Figure 1D) exhibited more vigorous growth and more intense green coloration compared to other treatments (Figure 1A-C). Plants watered with SSAM+molasses mixture showed a tendency toward more developed lateral roots and apparent lignification at the root base. Moreover, these plants appeared to have a greater number of branches and leaves compared to plants treated with molasses or SSAM solution alone. Control plants displayed visibly paler and smaller leaves compared to those treated with SSAM+molasses and all treated variants.
